# PATCH TESTING IN CHRONIC URTICARIA

**DOI:** 10.4103/0019-5154.53177

**Published:** 2009

**Authors:** Kiran V Godse

**Affiliations:** *Shree Skin Centre, 22 L Market, Sector 8, Nerul, Navi Mumbai - 400 706, India. Email: drgodse@yahoo.co.in*

Sir,

This is with reference to the article entitled “Use of patch testing for identifying allergen causing chronic urticaria” which appeared in the March–April issue of IJDVL.[1] The article illustrates the use of patch testing in the diagnosis of chronic urticaria. Author found 11 patients out of 57 with positive patch test. On avoidance of allergens, nine out of 11 patients showed complete disappearance of urticaria. All 11 patients experienced quick relapse after returning to normal lifestyle and diet. Author fails to mention the duration of chronic urticaria and severity of urticaria. The urticaria activity score consist of the sum of the wheal number score and the itch severity score.[2] The wheal numbers are graded from 0 to 3 as follows: 0 – less than 10 small wheals (diameter, <3cm); 1 – 10 to 50 small wheals or less than 10 large wheals (diameter, > 3 cm); 2 - greater than 50 small wheals or 10–50 large wheals; and 3 - almost the whole body is covered. The severity of the itching is graded from 0 to 3 (0, none; 1, mild; 2, moderate; and 3, severe). This scoring system is often used in the assessment of urticaria activity. Author mentions urticaria reduced to half in the progress report, which is difficult to understand. A control group without chronic urticaria was not included in the study.

None of the patients underwent autologous serum skin test, which can be done with the help of pathology laboratory. Mamatha, *et al.* and Bajaj, *et al.* mentions about positive autologous serum skin test in 34 and 49.5% of patients, respectively.[3,4] Greaves and Kaplan demonstrated the presence of circulating histamine–releasing factors in a proportion of patients with CU. Such factors were subsequently characterized as functionally active IgG autoantibodies specific for the high-affinity IgE receptor, FcEpsilon RI, or for IgE, able to induce degranulation of mast cells and basophils.[5,6] Autologous serum skin test (ASST) is a simple test to detect autoreactivity *in vivo* in patients with chronic urticaria. ASST is the only practicable test available to clinicians to detect autoimmune urticaria in India.[7] The real challenge is to find out the cause of remaining 50% patients who are ASST negative. Before the advent of ASST, chronic urticaria was considered as a mysterious disease possibly caused by anxiety and intolerance to foods, food dyes, or food additives. Many patients were advised restrictive diets for the treatment of urticaria. Similar views are expressed by Asero *et al.*, in the Blue journal.[8]

Observations of Sharma are of interest but need to be confirmed by other controlled studies before routine patch testing and avoidance measures are recommended in the diagnosis and treatment of chronic urticaria.

## References

[1] Sharma AD (2008). Use of patch testing for identifying allergen causing chronic urticaria. Indian J Dermatol Venereol Leprol.

[2] Erbagci Z (2002). The leukotriene receptor antagonist montelukast in the treatment of chronic idiopathic urticaria: A single-blind, placebo-controlled, crossover clinical study. J Aller Clin Immunol.

[3] George M, Balachandran C, Prabhu S (2008). Chronic idiopathic urticaria: Comparison of clinical features with positive autologous serum skin test. Indian J Dermatol Venereol Leprol.

[4] Bajaj AK, Saraswat A, Upadhyay A, Damisetty R, Dhar S (2008). Autologous serum therapy in chronic urticaria: Old wine in a new bottle. Indian J Dermatol Venereol Leprol.

[5] Greaves MW (2000). Chronic urticaria. J Allergy Clin Immunol.

[6] Kaplan AP (2002). Chronic urticaria and angioedema. N Engl J Med.

[7] Godse KV (2004). Autologous serum skin test in chronic idiopathic urticaria. Indian J Dermatol Venereol Leprol.

[8] Asero R, Cugno M, Tedeschi A (2007). Chronic urticaria: One step forward and two steps back. J Am Acad Dermatol.

